# Pragmatic controlled trial of a school-based emotion literacy program for 8- to 10-year-old children: study protocol

**DOI:** 10.1186/s12888-024-05628-z

**Published:** 2024-04-12

**Authors:** Alison L. Calear, Emily Macleod, Ashley M. Hoye, Sonia McCallum, Alyssa Morse, Louise M. Farrer, Philip J. Batterham

**Affiliations:** Centre for Mental Health Research, College of Health and Medicine, The Australian National University, Canberra, Australia

**Keywords:** Mental health, Health literacy, Child & adolescent psychiatry, Schools, Health education

## Abstract

**Background:**

Mental disorders are common in childhood, but many young people do not receive adequate professional support. Help-seeking interventions may bridge this treatment gap, however, there is limited research on interventions for primary-school children. This study aims to evaluate the effectiveness of an emotion literacy program at increasing literacy, reducing stigma, and promoting help-seeking in children aged 8–10 years.

**Methods and analysis:**

A two-arm pragmatic cluster-controlled trial will compare Thriving Minds, an emotion literacy program for middle primary school children, to a wait-list control condition. Children aged 8–10 years will be recruited from approximately 12 schools (6 intervention schools/6 wait-list control) to participate in Thriving Minds via direct invitation by the program delivery service. Allocation to the intervention condition will be pragmatically, by school. Children will receive the intervention over two 50-minute sessions, across two weeks. Using story books and interactive discussion, the program aims to develop children’s knowledge of their own and other’s emotional experiences and emotion regulation strategies (self-care and help-seeking). The primary outcome is help-seeking intentions. Secondary outcomes include help-seeking knowledge, attitudes, and behaviours, emotion knowledge and attitudes, and stigma. Children will complete surveys at pre-intervention, post-intervention (one week after the program) and 12-week follow-up. Additional satisfaction data will be collected from teachers in intervention schools via surveys (post-intervention and 3-month follow-up) and semi-structured interviews (after follow-up), and selected children via focus groups (12-week follow-up). Analyses will compare changes in help-seeking intentions relative to the waitlist control condition using mixed-model repeated-measures analyses to account for clustering within schools.

**Discussion:**

With demonstrated effectiveness, this universal emotion literacy program for promoting help-seeking for mental health could be more widely delivered in Australian primary schools, providing a valuable new resource, contributing to the mental health of young people by improving help-seeking for early mental health difficulties.

**Trial registration:**

Australian New Zealand Clinical Trials Registry, ACTRN12623000910606 Registered on 24 August 2023.

**Supplementary Information:**

The online version contains supplementary material available at 10.1186/s12888-024-05628-z.

## Strengths and limitations of this study

• This will be one of the first controlled trials of an emotion literacy program addressing mental health literacy, stigma, and help-seeking for mental health difficulties with primary school children aged 8 to 10 years.

• Quantitative measures, including predictors and mediators, are included in the study to enable statistical changes in outcomes to be assessed, as well as qualitative measures to explore participant experiences of using the intervention in greater depth.

• Limitations include the narrow age band, as further work may be needed to developmentally tailor the intervention for broader age groups.

• Parent feedback was not included due to study constraints (outside the intended focus of this research).

• Longer-term retention of knowledge and skills will not be assessed beyond 12-week follow-up and it is not the intention to assess impact on child mental health symptom outcomes.

## Introduction

Early access to mental health services and support is critical, as untreated mental disorders can significantly affect a child’s social, emotional and academic functioning, both in the short and long term [1]. Research suggests, however, that many young people do not readily seek or receive treatment or support for psychological distress or mental disorders [2, 3]. A recent systematic review and meta-analysis of the prevalence of mental disorders in children and adolescents in high-income countries found that only 44.2% of young people with mental disorders received any services for their conditions [2]. Another study conducted in Australia reported slightly higher rates of service use among children and adolescents, with 56.0% accessing services for emotional and behavioural problems [3].

Several barriers to help-seeking have been identified, including limited mental health knowledge (literacy), perceived social stigma and embarrassment, low perceived confidentiality and trust in mental health service providers, financial costs, logistical barriers, and limited availability of services [1]. Mental health knowledge and stigma are two modifiable factors that could be readily targeted in a low-intensity intervention to improve help-seeking in children. Schools have been identified as the ideal setting in which to implement mental health prevention programs including mental health literacy interventions, as they have unprecedented contact with children, and may be more likely to reach young people from marginalised backgrounds who often experience higher rates of mental disorders [4]. Indeed, some schools implement whole-school mental health prevention programs that may include elements of mental health literacy (e.g., KidsMatter [5]), with studies suggesting that these comprehensive programs can have positive effects on children’s mental health [6, 7]. We know less about brief, standalone mental health literacy interventions designed to facilitate help-seeking and reduce stigma, particularly for younger children.

To date, a number of studies have been conducted in schools to assess the effectiveness of brief interventions specifically designed to increase mental health literacy to improve help-seeking, and/or reduce stigma. A recent review of such interventions identified significant positive effects on knowledge and awareness of mental disorders in 86% of studies, and more positive attitudes and beliefs regarding mental disorders observed in 56% of studies [4]. Of the 22 randomised controlled trials (RCTs) identified in the review, only two were conducted with primary school (elementary) aged children.

The lack of rigorous research on mental health literacy programs in primary school aged children suggests an area of significant unmet need, particularly given evidence that mental health stigma can develop early [8, 9]. As such, educating children about mental health before their conceptualisations are fully formed may assist in preventing the formation of negative attitudes and foster more accurate knowledge and awareness of their own and other people’s mental health [10]. The two interventions delivered in primary schools that were identified in our review [11, 12] both targeted mental health stigma and reported positive effects on knowledge, attitudes and social distance. These studies were limited to the northern hemisphere, and only one was controlled with follow-up [11].

One of the challenges associated with delivering mental health literacy programs in primary school settings is that primary school age children have varying and emerging levels of emotion literacy, which is a prerequisite to mental health literacy. Jorm et al. [13] defined mental health literacy as “the knowledge and beliefs about mental health problems that help in their recognition, management, and prevention.” Here, drawing from literature on emotion understanding, we define *emotion literacy* as children’s conceptual knowledge and understanding of emotional experiences (including context, causes, physiological reactions, cognitions, and behaviours), and actions or responses that support regulation [14–16]. Lower emotion literacy is related to children experiencing anxiety, depression, and behavioural issues [17–19]. Emotion literacy is one of the main components of emotion competence [20, 21] and therefore a foundational part of mental health literacy. Understanding and differentiating between emotional experiences contributes to the ability to choose appropriate regulation strategies, including help-seeking.

Although children often require adult assistance to seek help, providing them with the capability to identify their experiences, knowledge of available supports, and the ability to destigmatise difficult emotional experiences, may facilitate direct help-seeking behaviour (e.g., through school psychologists) and indirect help-seeking (e.g., by giving them the language to tell a trusted adult about their mental health experiences). One newly established brief intervention promoting these skills is the Thriving Minds program for 8-10-year-olds [22]. Through story books and interactive discussion, the program aims to develop children’s knowledge of their own and other’s emotional experiences and emotion regulation strategies of self-care and help-seeking. The middle primary age group was chosen because it reflects a stage of childhood emotion development by which basic emotion knowledge has usually been established [23, 24]. From about 8 years old, children are developing a more complex understanding and knowledge of emotions, increasing their emotion regulation strategies, and learning about the moral components of emotion [14, 23, 24]. By early adolescence, children have developed an increasing awareness of social expectations of peers and others, enabling the awareness and endorsement of stigma about mental health; such stigma prevents help-seeking [25]. Given that stigma prevents help-seeking, middle primary years may be an ideal target age for interventions focusing on early de-stigmatisation and help-seeking for emotional experiences.

An uncontrolled post-program evaluation of the Thriving Minds program with two schools found a high level of satisfaction and engagement with the program, with 89% of students rating it 4.54 (*SD* = 0.76) stars out of 5. At the 6–8-week follow-up, over 85% of participants reported that they knew where to go to for help for difficult emotions [26]. The proposed multi-site pragmatic controlled trial will provide a more rigorous evaluation of the program in a broader sample of schools.

### Aims and hypotheses

Using a pragmatic cluster-controlled trial, this study will investigate the efficacy of a school-based mental health literacy program for 8-10-year-old children. The primary aim of the trial is to evaluate the effect of the Thriving Minds program on help-seeking intentions for uncomfortable emotions at post-intervention (and, secondarily, 12-week follow-up), compared to the wait-list control condition. It is hypothesised that participants receiving the Thriving Minds program, relative to participants in the wait-list control condition, will report higher levels of help-seeking intentions for uncomfortable emotions at post-intervention.

The secondary aims are to evaluate at post-test and 12 week follow-up the following: 1) the efficacy of Thriving Minds on a range of help-seeking outcomes: (a) help-seeking confidence, b) help-seeking knowledge, c) help-seeking attitudes, d) actual self-care behaviours, and e) actual help-seeking behaviours; 2) the effect of Thriving Minds on emotion knowledge ((a) emotion recognition and (b) physiological components of emotion); and 3) the effect of Thriving Minds on stigma. It is hypothesised that participants receiving Thriving Minds will report improvements in each of these outcomes relative to the waitlist control.

Exploratory aims include exploring *Predictors and mediators* of intervention effects, including demographic characteristics, mental health conditions, emotion knowledge (emotion vocabulary, and bodily awareness of emotions) and emotion stigma (emotion attitudes (not hiding emotions)), wellbeing, and school support. These analyses will also investigate subgroup effects, such as whether the Thriving Minds program has a greater effect on emotion knowledge among students with low pre-existing knowledge.

A final aim of the study is to conduct a qualitative evaluation to assess *student and staff satisfaction* with the program, including what students liked and learned in the program, any suggested improvements, and any observable changes in student learning, behaviour or wellbeing following the intervention.

## Methods and analysis

### Study design

This study protocol complies with the SPIRIT guidelines (Supplementary Materials B). A pragmatic controlled cluster trial will be conducted in at least 12 primary schools (6 intervention condition/6 wait-list control condition) with three measurement occasions (pre-intervention, post-intervention and 12-week follow-up). Surveys for participating classroom teachers will be delivered at post-intervention and 12-week follow-up. A qualitative evaluation will also be conducted through open-ended survey items for students at post-intervention, focus groups with selected children, and semi-structured interviews with selected staff from intervention condition schools after the12-week follow-up assessment.

### Participants and recruitment

We will recruit approximately 500 8- to 10-year-old students in total, drawn from at least 12 schools in the Australian Capital Territory (ACT). Based on a previous primary school RCT of a mental illness stigma intervention [11], we anticipate an effect size of *d* = 0.35 in literacy and stigma. Based on typical session sizes of *n* = 40–50 students per school, recruiting at least 12 schools (6 intervention/6 control; N = approx. 500) will provide > 83% power to detect an effect, assuming up to 20% attrition and accounting for clustering with an intraclass correlation of 0.03. Schools will be recruited via pre-existing relationships with the intervention provider, MIEACT. All children in participating school year groups will be eligible to participate, pending parental consent. Information and consent forms will be distributed by classroom teachers to students and their parent/guardian prior to the trial commencing. Children with parental consent will also receive study information and provide written consent at the time of their first survey and will assent via survey completion for subsequent surveys. All participants will have access to information on crisis support numbers and mental health websites for use if they are feeling distressed. This information will be included on the information sheet provided to participants and their parents/caregivers, as well as at the end of each survey.

Usual teachers of participating students will be asked to complete a brief survey at post-intervention and 12-week follow-up timepoints (*n* = approximately 30). We aim to also invite between one and three school staff per intervention school to participate in a semi-structured interview after the 12-week follow-up survey. The staff members targeted for interviews will be Executive staff holding key pastoral care positions and classroom teachers.

### Condition allocation

Allocation of schools to the trial conditions (intervention or wait-list control) will be on a pragmatic basis, due to the limited flexibility in school timetables. Where possible, schools will be matched across conditions based on school type (public vs. private) and socioeconomic status, with at least six schools per condition. Using a predetermined schedule accounting for program delivery staff availability, MIEACT will provide schools with a choice of available times in which Thriving Minds could be delivered and schools will nominate feasible options based on timetable and curriculum planning. School nominations will contribute to their allocation to the intervention or control condition (e.g., schools who choose early year program delivery will need to be intervention schools, due to insufficient lead time for control condition surveys). Parents will be sent consent forms with random, unique participant ID numbers so that signing a consent form allocates each child a participant ID number, which will also allow the linking of parent provided child mental health information to each child.

### Procedure

**Fig. 1:**
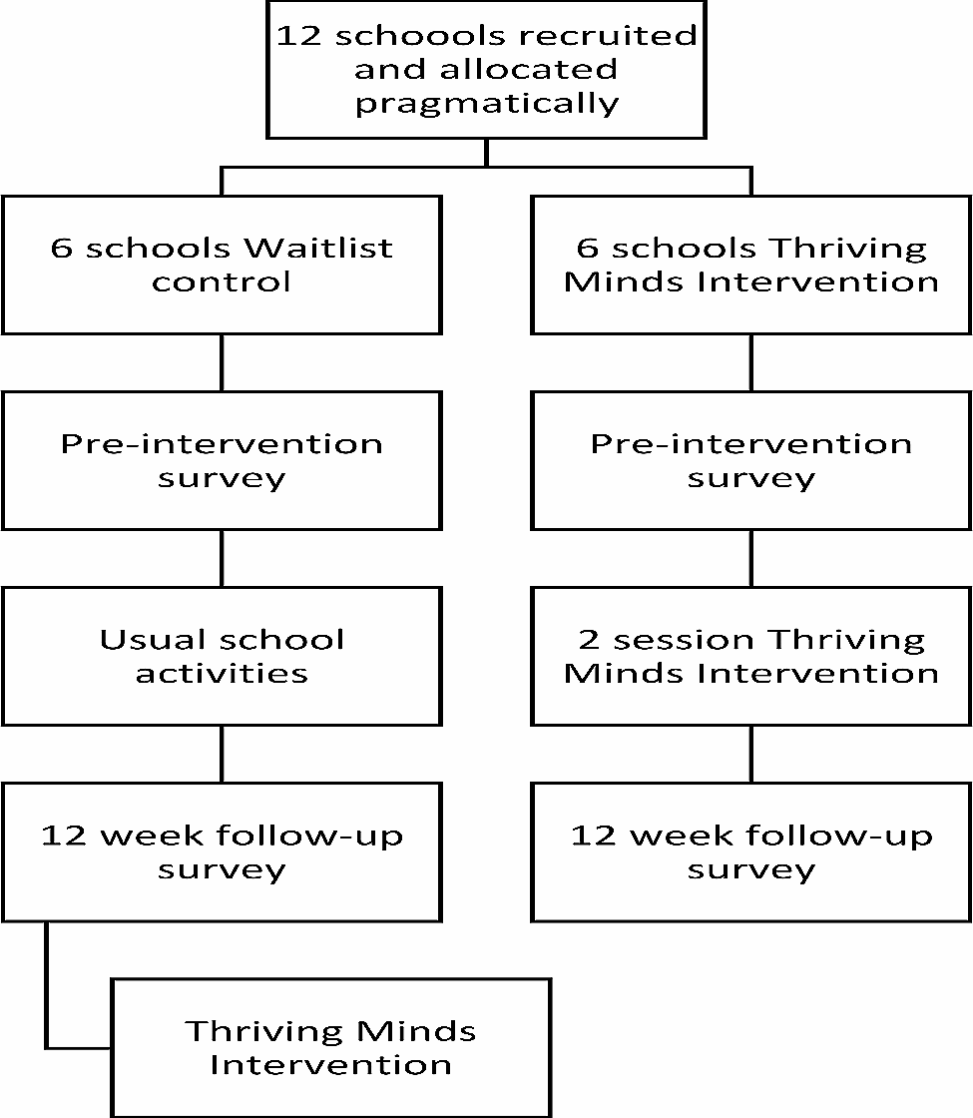
Flow of participants

To facilitate recruitment and intervention delivery, the trial will be conducted over at least two school years (Terms 3 and 4, 2023 and Terms 1 and 2, 2024). All consenting students will be invited to complete a pre-intervention survey one week prior to the intervention condition schools receiving the Thriving Minds program. The program will be delivered to all students in intervention condition schools over two weeks by trained educators. During this period, wait-list control condition schools will continue usual classroom activities. Following the intervention phase, all students will complete a post-intervention survey (one week after the intervention), followed by a third 12-week follow-up survey to assess longer-term intervention effects. All surveys will be completed by pencil and paper, or online using Qualtrics (online survey software) depending on the school’s preference. Researchers, usual classroom teachers, learning support staff, and usual school volunteers will assist children to complete the surveys (e.g., assist with reading survey items or clarifying instructions). Surveys will take approximately 30 min to complete. Teachers in participating classrooms in intervention condition schools will complete a program evaluation survey at the post-intervention and 12-week follow-up timepoints. Figure 1 presents the flow of the participants in the trial.

Students in the wait list control condition will receive the Thriving Minds program following the completion of the 12-week follow-up survey. After the final survey, semi-structured interviews (30–60 min) will also be conducted in intervention condition schools with staff members who are best placed to provide qualitative insights into cultural changes in the classroom and school and attitudinal or behavioural changes in the students who received the intervention. Focus groups (30–45 min) will also be conducted with children to explore their perspectives on the program. Clinical support will be provided if a participant becomes distressed while completing trial surveys or focus groups.

### Intervention

All students will receive the Thriving Minds program, either after the first survey (intervention condition), or after the third survey (wait-list control condition). The Thriving Minds program is conducted over two 50-minute sessions, delivered one week apart. The program aims to address mental health literacy and stigma by normalising uncomfortable emotional experiences, equipping students with effective self-care strategies, and encouraging them to seek help for emotional problems. Content is mapped to the Australian curriculum and delivered with classroom teachers present, who are ideally placed to support ongoing classroom use of emotion literacy content [27]. The program sessions focus on key concerns for 8–10-year-olds, including coping with stress/anxiety and low mood. The content is delivered through developmentally appropriate story books relating to anxiety and depression and classroom discussions and activities relating to the development of emotion literacy. Specifically, content focuses on recognition of emotional experiences (identification of different emotion characteristics, including physiological characteristics, distinguishing between comfortable and uncomfortable and big and small emotions), knowledge about appropriate regulation actions (self-care strategies), and help-seeking for uncomfortable emotions. A summary of the program content is included in the Supplementary Materials A.

### Patient and public involvement

Thriving Minds was developed by MIEACT in response to feedback from schools indicating a gap in mental health literacy education for 8-10-year-old children. School teachers were consulted in the design of the program and provided feedback in the uncontrolled post-program evaluation to improve the design. For the current evaluation, prior to the commencement of the trial, a sample of 8–10 year-old children trialled the measures. Adjustments were made based on their responses to improve clarity and comprehension. School education directorates assessed and approved the burden of the intervention and time required to participate in the research and approved measures. Schools will be provided with study summary findings to disseminate to their communities.

### Measures

Table 1 presents the proposed measures that will be administered to children, teachers, and parents at each measurement occasion in the Thriving Minds trial. Some measures are existing validated measures that have been adapted for developmental literacy levels as indicated, and other measures were developed specifically for the study where no existing validated measures were available for children.

#### Help-seeking

The primary outcome for the current trial is *Help-seeking intentions* and will be assessed using the General Help-Seeking Questionnaire [GHSQ] [28]. Participants will be asked to rate how likely they are to seek help for big uncomfortable emotions from a range of people, including a friend, teacher, parent, family member, psychologist or school counsellor, doctor, and Kids Helpline (phone helpline). Participants will be provided with the opportunity to nominate another person (‘Other’) if they want to. All items will be responded to on a 5-point scale ranging from 1 (Extremely unlikely) to 5 (Extremely likely). Participant *help-seeking knowledge* will be measured by asking children to provide a free text response to indicate who they would get help from if they were having uncomfortable emotions (adapted to an open-ended question from a multi-choice response item on the GHSQ [28]). Total informal (e.g., family, friends, teacher) and formal (e.g., GP, psychologist, helpline) sources of help will be calculated.

Participant *help-seeking confidence* will be measured with a single item asking children to indicate on a 5-point scale ranging from 0 (Not at all) to 4 (Extremely) how easy it is for them to seek help. This item is adapted from a previous study [29], with the term ‘easy’ replacing ‘confidence’ to align with developmental literacy levels.

Participant *help-seeking attitudes* will be measured by asking children to rate how helpful each of 12 actions are for when they have *big, uncomfortable emotions*, using a 5-point rating scale ranging from 1 (very unhelpful) to 5 (very helpful), adapted from [30]. The list includes *no action* options (e.g., ‘Pretend the emotions aren’t there’), *self-help* actions aligning with the content of the Thriving Minds program (e.g., ‘Do something to make myself feel better (self-care)’), *informal help-seeking* actions (e.g., ‘Talk to a parent’) and *formal help-seeking* actions (e.g., ‘Talk to a psychologist or school counsellor’). Each individual response item will receive a helpfulness rating, and average scores across no action, self-help, informal help-seeking and formal help-seeking will also be calculated.

*Actual Help-Seeking and Self-Help Behaviours* will be measured using an adapted version of the Actual Help-Seeking Questionnaire (AHSQ) [31]. The list of response options will be the same as the *attitudes to help-seeking* question (see above), with participants endorsing the sources/activities they have engaged with. Total ‘no action’, ‘self-help’, *‘*informal help-seeking’, and ‘formal help-seeking’ scores will be calculated.

#### Emotion Knowledge

Participants will demonstrate their *emotion vocabulary* by making a list of comfortable and uncomfortable emotions (similar to [32, 33]). The total number of emotions that participants list and correctly categorise as comfortable and uncomfortable will be counted.

Participant *emotion recognition* will be measured using three vignettes about children having uncomfortable emotional experiences that were developed to align with content covered in the Thriving Minds program (exclusion from a birthday party, missing out on a favourite breakfast cereal, and worry about an upcoming school carnival). Children will be asked to identify the emotion(s) that they think the character is feeling from a list of 12 emotions (frustrated, worried, depressed, relieved, happy, sad, relaxed, angry, anxious, bored, disappointed, scared). Children will also be asked to identify if the character’s emotion is comfortable, uncomfortable, or not sure, and big, small, or not sure. Total appropriate, potentially appropriate, and inappropriate emotions will be coded and calculated for each vignette, and average scores across the three vignettes will also be calculated. Average accuracy of identification of comfort and size of emotions will also be calculated.

Participants will be asked about their knowledge of the *physiological components of emotions* by indicating “which feelings occur in the body when someone is feeling sad/happy/worried?” Children will be able to select one or more options from a list of 13 options (e.g., Hot, Calm, Beating Heart). ‘Not sure’ and ‘Other’ options will also be available. The question was developed to align with Thriving Minds content. Total appropriate, potentially appropriate, and inappropriate physiological components will be coded and calculated for each vignette, and average scores across the three vignettes will also be calculated.

Participant *bodily awareness of emotions* will be measured using the 5 items of the Bodily Awareness sub-scale of the Emotion Awareness Questionnaire [EAQ], α = 0.64 [34]. Each item has a 3-point response scale ranging from 1 (Not true) to 3 (True). A mean sub-scale score will be calculated across items, with scores ranging from 6 to 18. Items are reverse coded (except one) so that higher scores reflect lower bodily awareness.

#### Emotion attitudes

Children’s *emotion attitudes* will be measured using the 5 items of the Not Hiding Emotions subscale of the EAQ, α = 0.68 [34]. Each item has a 3-point response scale ranging from 1 (Not true) to 3 (True). All items are reverse scored. A mean sub-scale score will be calculated across items, with scores ranging from 6 to 18. Higher scores reflect less hiding of emotions.

#### Stigma

Participants will be provided with two vignettes, one about worry, and one about sadness, adapted for developmental appropriateness (less text, simpler language) from vignettes about anxiety and depression that have been used in prior research with adolescents [35]. *Stigma attributions*, or negative judgement pertaining to children who experience uncomfortable emotions, will be measured by asking participants to indicate how much they agree or disagree with six statements about each vignette character, using a 7-point scale ranging from 1 (Strongly disagree) to 7 (Strongly agree). All items were adapted from the Revised Attribution Questionnaire [r-AQ], α = 0.68 [36, 37]. A total stigma attribution score will also be calculated by summing the item scores. Total scale scores can range from 6 to 42, with higher scores indicative of higher stigmatising attitudes. An additional item (“I would not want to be friends with [character]”) was also added to the scale, adapted from [35], which will be analysed individually.

Emotion stigma will also be measured by asking participants to complete a *stigma association* task adapted for developmental appropriateness (less options, simpler language) from the Stigma of Suicide Scale Short Form (SOSS-SF) [38]. Participants will be provided with the statement “people who have big feelings are” and ask them to rate using a 5-point scale ranging from 1 (Strongly disagree) to 5 (Strongly agree) their level of agreement with a list of 15 descriptors (e.g., attention-seeking, brave, embarrassing, hurtful to others, lazy). Items load onto 3 subscales (Stigma, α = 0.88; Isolation/depression, α = 0.80; Normalisation/glorification, α = 0.78). Mean subscale scores will be calculated, ranging from 1 to 5. Higher scores reflect higher levels of stigma, greater attribution to isolation/depression or greater normalisation/glorification.

#### School support

School support will be measured using two scales originally from the Sources of Strength trials [39, 40], adapted with minor language modifications. Four items will be used to assess *peer integration* at school, addressing both inclusion and isolation. Participants will respond to each item on a 4-point scale ranging from 1 (Strongly disagree) to 4 (Strongly agree). A mean scale score is calculated. Higher scores reflect higher peer integration. *Trusted adults at school* will be measured using a 4-item scale, α = 0.90 [41] on which participants rate their connections to adults at school on a 4-point scale ranging from 1 (strongly disagree) to 4 (strongly agree). A mean scale score is calculated, with higher scores indicative of students having adults at school that they feel they can trust and talk to about problems.

#### Wellbeing

Using the Stirling Children’s Wellbeing Scale (excluding the 4 lie-scale items) [42], participants will be asked to rate 12 statements about their mood, levels of enjoyment, and positive expectations in life, using a 5-point scale ranging from 1 (Never) to 5 (All the time), α = 0.82. Total scale scores are calculated by summing item scores, with total scale scores ranging from 12 to 60. Higher scores are reflective of greater emotional wellbeing.

#### Demographic and study characteristics

In the pre-intervention survey, all participants will be asked to provide their age (7, 8, 9, or 10 years) and gender (boy, girl, don’t feel like a boy or girl). At post-intervention, participants in the intervention condition will be asked to indicate the number of Thriving Minds sessions they completed (0, 1, 2, unsure). At the time of consent, parents will be asked to indicate if the participating child speaks more than one language, and if the child has a diagnosis of autism, attention-deficit/ hyperactivity disorder (ADHD), learning difficulties, depression, anxiety, or another mental health condition. Parents can elect not to complete these items. School decile ratings will be used to provide an indication of socio-economic status.

#### Acceptability and Satisfaction

Program acceptability and satisfaction will be assessed in intervention condition schools, at post-intervention using a 3-item bespoke measure and open-ended questions assessing what participants liked most and least about the program and what they found to be most helpful.

### Teacher survey

Usual classroom teachers at intervention schools will be asked to complete a brief survey at the post-intervention and 12-week follow-up timepoints. At each time-point, teachers will be asked to rate the extent to which they agree with nine statements regarding perceived value of program content, intention to use content, and actual use of content, using a 5-point scale ranging from 1 (Strongly disagree) to 5 (Strongly agree). A sixth response option, ‘NA/not sure’, will also be available. Teachers will also be asked four open-text response questions identifying what they liked most and least about the Thriving Minds program, possible program improvements, and if they had completed any follow-up activities relating to Thriving Minds content.

### Focus groups and interviews

Two groups of 4–5 children from 3 to 4 intervention condition schools will be invited to take part in focus groups to further explore the value, strengths and weakness of the Thriving Minds program. Using open-ended prompts, children will be asked to discuss what they learned from the program, its strengths and weaknesses, how ideas from the program have been used in the classroom or school environment, what they told their parents about the program, and what they would tell a peer about the program.

Semi-structured interviews will also be conducted with teachers and executive staff responsible for student wellbeing in intervention condition schools at 12-week follow-up.

**Table 1:** Intervention and measures timeline

	Study Period				
	Pre-intervention	Intervention	Post-intervention	12-week follow-up	Post 12 week follow-up
**Interventions**					
Intervention					
Waitlist Control					
***Child Measures***					
**Demographic Characteristics**					
Age	✓		✓	✓	
Gender	✓		✓	✓	
Languages spoken (parent reported)	✓				
Mental health diagnosis (parent reported)	✓				
**Help-seeking Measures**					
Help-seeking knowledge, Adapted GHSQ [13]	✓		✓	✓	
Help-seeking intentions, GHSQ [28]	✓		✓	✓	
Help-seeking confidence [29]	✓		✓	✓	
Help-seeking attitudes, adapted from [30]	✓		✓	✓	
Actual self-help behaviours, Adapted AHSQ [31]	✓		✓	✓	
**Emotion Knowledge**					
Emotion Vocabulary, bespoke measures adapted from [32, 33]	✓		✓	✓	
Emotion Recognition (bespoke measure)	✓		✓	✓	
Physiological components (bespoke measure)	✓		✓	✓	
Bodily Awareness: EAQ subscale [34]	✓		✓	✓	
**Emotion Attitudes**					
Not Hiding Emotions: EAQ subscale [34]	✓		✓	✓	
**Stigma**					
Emotion stigma: Vignette rating scale, adapted from [35–37]	✓		✓	✓	
Emotion Stigma: Adapted SOSS [38]	✓		✓	✓	
**School support**					
Trusted adults at school [39, 41]	✓		✓	✓	
Peer integration at school [39]	✓		✓	✓	
**Wellbeing**					
Stirling Children’s Wellbeing Scale [42]	✓		✓	✓	
**Acceptability and Satisfaction**					
Acceptability and Satisfaction (bespoke measure)			✓	✓	
***Teacher Measures***					
Perceived value, intention to use, actual use (bespoke survey measure)			✓	✓	
***Qualitative measures***					
Staff interviews				✓	
Child focus groups				✓	

Teachers will be asked to share their perspectives on the program, including its acceptability, overall satisfaction with program content, and any observed changes in student behaviour and/or school culture that may have occurred as a result of the Thriving Minds program.

### Data analysis plan

Written survey data will be entered verbatim into Qualtrics by researchers who are blind to participant condition. All research data will be securely stored at the Australian National University for at least five years from the date of any publication arising from the research and will be accessible to members of the research team. At the end of the five-year period, electronic survey and transcription data will be archived in a deidentified format (all reasonably identifying information removed) and may be shared with other researchers, with permission from the original research team.

Analyses of continuous measures will be undertaken on an intention-to-treat basis, including all participants allocated regardless of treatment actually received or withdrawal from assessments. Mixed-model repeated measures (MMRM) analyses will be used because of the ability of this approach to include participants with missing data. In addition, by incorporating appropriate random effects for each school, MMRM will accommodate and assess the strength and significance of clustering effects. MMRM is the standard and most robust methodology for analysing cluster randomised trials [43, 44]. Using (MMRM) ANOVA models, measurement occasion will be included as the within-group factor and condition as the between-groups factor [45]. For any dichotomous outcomes, a comparable binary mixed modelling approach [46] will be used. Cohen’s *d* effect sizes will be calculated for primary and secondary outcomes at each time-point. If efficacy is demonstrated, exploration of potential mediators and moderators of response, such as child age, child mental health, baseline emotion literacy, participant gender and intervention completion, will be explored separately using 3-way interaction terms subgroup analyses.

Qualitative analysis of the semi-structured interviews will be conducted using Framework Analysis. Framework Analysis was designed for addressing social policy research questions [47] and has been used frequently in health research [48]. It follows a systematic process of inductive coding, generating a table of findings across participants that is conducive to interpretation and input from multiple researchers.

## Discussion

Results of the current study will be communicated in aggregate form to key stakeholders, parents/carers, mental health practitioners, education providers, and the academic community through community forums, academic conferences and peer-reviewed publications.

The proposed study will be one of the first rigorous evaluations of a mental health awareness program in Australian primary schools that targets mental health literacy, stigma and help-seeking for mental health difficulties. There is a clear need for evidence for such interventions in children given the growing prevalence of mental disorders in this age group. The results of the proposed study will provide vital new evidence on the effectiveness and acceptability of mental health awareness interventions in this population.

The proposed study will provide important new knowledge on the effectiveness and acceptability of mental health awareness programs in children. If found to be effective, the Thriving Minds program could be developed to include a wider developmental age-group, integrated within the school curriculum, and scaled up for delivery across Australia, providing a low-intensity and accessible intervention to promote mental health and timely help-seeking in our children, now and into the future. Timely access to appropriate services and support for mental disorders is essential, as it is associated with improved treatment outcomes and can reduce the social, emotional, and academic difficulties that can occur as a result of untreated mental disorders [1]. Although the success of mental health education relies on the right services and supports being available, promotion of mental health awareness, de-stigmatisation, and help-seeking are important first steps in mental health intervention.

### Electronic supplementary material

Below is the link to the electronic supplementary material.

**Supplementary Material A:** Thriving Minds Intervention Content

**Supplementary Material B:** SPIRIT Guidelines

## Data Availability

No datasets were generated or analysed during the current study.
