# Supplementary material for: Pragmatic controlled trial of a school-based emotion literacy program for 8- to 10-year-old children: study protocol
Source: BMC Psychiatry. 2024 Apr 12;24:275. doi: 10.1186/s12888-024-05628-z (PMC11010293; doi:10.1186/s12888-024-05628-z)
Supplement: Supplementary file 1 — Supplementary Material A: Thriving Minds Intervention Content [file 12888_2024_5628_MOESM1_ESM.docx]

### Supplementary Materials A: Thriving Minds Intervention Content

| Session One | |
| --- | --- |
|  | Acknowledgement of Country, introduction, and establishment of class rules |
| Activity One:  Discussion | - Naming emotions - Recognising physical symptoms of emotions. - Emotions as uncomfortable or comfortable, big and small - Importance of being able to identify and talk about emotions. |
| Activity Two: Children’s storybook: Princess and the Fog (1) | - Book content includes emotions such as sadness and depression, and help-seeking behaviours - Discussion involving identifying character’s emotions and help-seeking behaviours. |
| Activity Three: Self-care | - Discussion of concept of creating and using a personalised self-care box to help with uncomfortable emotions |
| Activity Four: Help-seeking | - Identification of people and services children can go to for help with emotions (aiming for a minimum of 5). |
| Activity Five: Mindfulness exercises | - Mindfulness exercises |
| Session 2 | |
|  | Acknowledgement of Country and recap from last week |
| Activity One: Window of Tolerance | - Conceptualising emotions as tolerable/manageable, elevated and associated with hyperarousal, or low/associated with hypoarousal - Practice recognising emotions and their relation to the window of tolerance. - Connect self-care and help-seeking to uncomfortable emotions in the window of tolerance |
| Activity Two: Children’s storybook: Ruby’s Worry (2) | - Book content includes emotions relating to anxiety and bravery - Discussion involving identification of character's emotions and connection to the Window of Tolerance |
| Activity Three | - Emotion guessing game (charades) |
| Activity Four: Children’s storybook: Strictly No Elephants (3) | - Book content includes emotions relating to social exclusion and inclusion - Discussion relating to identification of character’s emotions and noticing and helping others with their emotions |
| Activity Five: Mindfulness exercises | - Mindfulness exercises |

1. Jones L. The Princess and the Fog: Jessica Kingsley Publishers; 2015.

2. Percival T. Ruby's Worry: Bloomsbury Children's Books; 2018.

3. Mantchev L, Yoo T. Strictly No Elephants: Simon & Schuster Books for Young Readers; 2015.
